# A combined genetic and phenotypic marker approach enables precise detection of hypervirulent *Klebsiella pneumoniae* and reveals associated traits of capsule overproduction and tellurite resistance

**DOI:** 10.1128/spectrum.02474-25

**Published:** 2026-01-07

**Authors:** Tran Thi Thuy Duong, Wei-Hung Lin, Ya-Min Tsai, Chun-Hsing Liao, Yun-Tsung Huang, Po-Ren Hsueh, Cheng-Yen Kao

**Affiliations:** 1 Institute of Microbiology and Immunology, College of Life Sciences, National Yang Ming Chiao Tung University 34914, Taipei, Taiwan; 2 Division of Nephrology, Department of Internal Medicine, National Cheng Kung University Hospital, College of Medicine, National Cheng Kung University 63461, Tainan, Taiwan; 3 Department of Clinical Laboratory, En Chu Kong Hospital, New Taipei City, Taiwan; 4 Division of Infectious Diseases, Far Eastern Memorial Hospital 46608, New Taipei City, Taiwan; 5 Department of Laboratory Medicine and Internal Medicine, National Taiwan University Hospital, National Taiwan University College of Medicine 38006, Taipei, Taiwan; 6 Departments of Laboratory Medicine and Internal Medicine, China Medical University Hospital, China Medical University 38020, Taichung, Taiwan; 7 Ph.D. Program for Aging, College of Medicine, China Medical University, Taichung, Taiwan; 8 Health Innovation Center, National Yang Ming Chiao Tung University 34914, Taipei, Taiwan; 9 Microbiota Research Center, National Yang Ming Chiao Tung University 34914, Taipei, Taiwan; Zhejiang University, Hangzhou, China

**Keywords:** capsule, carbapenem-resistant, hypervirulent *Klebsiella pneumoniae*, liver abscess, tellurite resistance

## Abstract

We integrated genetic and phenotypic analyses to characterize carbapenem-resistant hypervirulent *K. pneumoniae* (CRhvKp) and liver abscess-associated *K. pneumoniae* (LAKp) isolates, aiming to identify potential biomarkers for the accurate detection of hvKp. A total of 660 carbapenem-resistant *K. pneumoniae* (CRKp) and 69 LAKp isolates were screened for five hvKp-associated biomarkers (*peg-344*, *iroB*, *iucA*, *_p_rmpA*, *_p_rmpA2*) and the string test. Among LAKp isolates, 49.3% were positive for all five biomarkers and the string test (5 genes-string test positive), while 39.1% carried all markers but were string test-negative (5 genes-string test negative). The seven-day post-infection survival rates of larvae infected with 5 genes-string test positive, 5 genes-string test negative, and other LAKp isolates were 5.9%, 10.4%, and 4.3%, respectively. In contrast, larvae infected with CRKp isolates showed higher survival rates of 7.1%, 17.9%, and 37.5% for the other groups. NTUH-K2044 and the hypervirulent isolates CRKp3, CRKp117, and LAKp88 exhibited higher lethality in bacteremia mice model compared to CRKp isolates lacking five virulence genes and string test negative. Moreover, 5 genes-string test negative LAKp produced the highest levels of capsule (117.7 ng/10⁶ CFU), followed by 5 genes-string test positive LAKp (101.4 ng/10⁶ CFU). Notably, carbapenem-resistant classical *K. pneumoniae* (CRcKp) isolates lacking five virulence genes and string test negative, markedly lower capsule levels (7.4 ng/10⁶ CFU) compared to other groups. These results suggest that the combined use of the five genetic markers and the string test enables effective identification of hvKp, and the capsule production is correlated with the virulence potential of the strains.

**IMPORTANCE:**

This study tackles the growing threat of *K. pneumoniae* strains that combine high antibiotic resistance with hypervirulence. By integrating genetic and phenotypic markers, we examined carbapenem-resistant *K. pneumoniae* (CRKp) and liver abscess-associated *K. pneumoniae* (LAKp) isolates. We found strong associations between five key virulence genes (*peg-344*, *iroB*, *iucA*, *_p_rmpA*, *_p_rmpA2*), positive string test results, high capsule production, and tellurite resistance. A 23-year surveillance of 1,815 carbapenem-susceptible isolates revealed that the proportion of blood isolates carrying all five virulence biomarkers, regardless of string test positivity, was significantly higher than that of urine isolates. Our findings provide a practical framework to enhance detection and risk assessment of hypervirulent *K. pneumoniae*, while deepening understanding of the traits that drive their clinical impact.

## INTRODUCTION

Hypervirulent *Klebsiella pneumoniae* (hvKp) was first reported in Taiwan in the mid-1980s and has been disseminated globally in recent years (1). hvKp differentiates from classical *Klebsiella pneumoniae* (cKp) in its ability to cause invasive infections even in healthy adults and is often associated with pyogenic liver abscess (LA) (2). On the contrary, cKp is an opportunistic pathogen that can cause infections primarily in healthcare settings in the elderly or in immunocompromised patients (3). Hypervirulence of hvKp is associated with the hypermucoviscosity phenotype determined by a positive string test and mediated by the presence of *magA* (mucoviscosity-associated gene A) and *_p_rmpA* and/or *_p_rmpA2* (regulators of the mucoid phenotype) genes with overproduction of capsular polysaccharide for immune evasion (4, 5). Furthermore, capsular serotypes (K antigen) K1 and K2 are dominant in hvKp (6). Compared to cKp, hvKp also produces more siderophores, such as salmochelin (*iroB* gene encoded) and aerobactin (*iucA* gene encoded), through the acquisition of virulence plasmids and, thus, has higher iron acquisition ability (3, 7).

Patients with hvKp invasive infections usually have poor outcomes due to the rapid progression of the disease. Therefore, biomarkers for precise and rapid diagnosis to detect hvKp infections are urgently needed in clinical settings. The string test, *Galleria mellonella* infection combined string test, tellurite resistance test, and siderophore production test have all been previously reported in the literature as methods for detecting hvKp, demonstrating varying levels of accuracy (90%–94.9%), specificity (89%–96%), and sensitivity (91%–100%) (8). Computational analysis revealed that tellurium gene clusters are commonly found in well-characterized hypervirulence plasmids of hvKp, such as pK2044 and pLVPK (8, 9). In contrast, the correspondence between the string test and the clinical features observed with hvKp infections is variable (10).

Russo et al. reported that virulence biomarkers, including *iroB*, *iucA*, *_p_*rmpA, *_p_*rmpA2, *peg-344* (encodes the transporter of metabolites), and *peg-589* (encodes the putative carboxymuconolactone decarboxylase family protein), accurately predict mortality in a murine sepsis model (10). However, studies investigating the prevalence of these biomarkers, their combinatorial patterns, and the interplay between specific biomarker combinations and phenotypic traits in relation to *K. pneumoniae* virulence remain limited. Furthermore, data on the distribution of these biomarkers across isolates from different clinical sources, such as blood, urine, and liver abscesses, are also relatively scarce. In this study, in addition to the traditional hypervirulent lineage-associated liver abscess *Klebsiella pneumoniae* (LAKp), we investigated the prevalence of hvKp in carbapenem-resistant *Klebsiella pneumoniae* (CRKp) and performed a comprehensive analysis of virulence and plasmids in carbapenem-resistant hypervirulent *Klebsiella pneumoniae* (CRhvKp) using phenotypic and genotypic biomarkers.

## MATERIALS AND METHODS

### Collection of *Klebsiella pneumoniae*

This study was approved by the institutional review board of the En Chu Kong Hospital (approved number: ECKIRB1111206), Far Eastern Memorial Hospital (approved number: 112074-E), National Taiwan University Hospital (approved number: 202112098RIND), and National Cheng Kung University Hospital (approved number: A-ER-112-213). A total of 322, 187, and 151 CRKp isolates were collected from patients in En Chu Kong Hospital (2011–2021, Northern Taiwan), National Taiwan University Hospital (2017–2020, Northern Taiwan), and National Cheng Kung University Hospital (1999–2020, Southern Taiwan), respectively. Sixty-nine *K. pneumoniae* isolates from LA patients collected from Far Eastern Memorial Hospital (29 isolates in 2007 and 40 isolates in 2017) were used as typical hvKp controls. In addition, 665 and 1,150 carbapenem-susceptible *K. pneumoniae* (CSKp) isolates recovered from patients with bloodstream infections and urinary tract infections, respectively, at National Cheng Kung University Hospital (from 1999 to 2022), were used to evaluate the performance of multiplex PCR screening tests and the prevalence of five genetic biomarkers. *K. pneumoniae* isolates were stored at −80°C in lysogeny broth (LB) containing 20% glycerol (vol/vol) until tested.

### String tests

*K. pneumoniae* isolates were cultured on blood agar plates overnight at 37°C. A 10 μL inoculation loop was used to touch the colonies and gently lift them for the string test (11).

### Capsular serotyping and MLST typing

Genomic DNA (gDNA) of isolates was extracted by boiling methods and tested by PCR for the presence of capsular serotype-specific (*cps*) genes (K1, K2, K5, K20, K47, K54, K57, and K64) (12). The primers used are listed in Table S1. The allele sequences (*gapA*, *infB*, *mdh*, *pgi*, *phoE*, *rpoB*, and *tonB*) were sequenced (13), and sequence types (STs) were determined by the Institut Pasteur Klebsiella MLST database.

### Multiplex PCR detection of five genetic biomarkers

The primer sets for multiplex PCR targeting hypervirulence-associated genes (*iroB*, *iucA*, *peg-344*, *_p_rmpA*, and *_p_rmpA2*) were designed, with the *iucA* primer adopted from previously published literature (10). The primer sequences and expected product sizes are listed in Table S1. The annealing temperature for multiplex PCR amplification was set to 59°C, and the reaction included 25 cycles of target amplification. PCR was conducted using the GeneExplore thermal cycler (Bioer Technology, China) with 96-well PCR plates (MB-P96-T and MB-PSM, Gunster Biotech, Taiwan). Agarose gels stained with ethidium bromide (EtBr) were used for visualization, and PCR products were examined under ultraviolet light.

### *Galleria mellonella* infection

The *G. mellonella* larva infection test was carried out as previously reported (14, 15). The larvae weighed around 240 mg were injected with a 10 µL bacterial suspension (2.5 × 10^6^ CFU) into the second to last left proleg, while the control larvae received the same volume of sterilized PBS. The larvae were kept at 37°C in the dark without feeding for 7 days, and survival was recorded every 24 h until 7 days after injection. Each set of 5 larvae received a 10 μL bacterial suspension injection, and larvae infection was performed in biological duplicates to ensure reproducibility (10 larvae/isolate).

### Mouse infection and bacterial load determination

The animal experiments were approved by the Institutional Animal Care and Use Committee (IACUC) of National Yang Ming Chiao Tung University (approved number: 1130431). A 100 μL suspension (10^4^ CFU/mL) was administered intraperitoneally to 7-week-old BALB/c male mice (sourced from National Laboratory Animal Center) using a 0.5 mL insulin syringe. Each isolate was used to infect six mice, and their survival was monitored over 7 days post-infection.

To assess bacterial colonization rates across different mouse organs, we infected six mice per strain and collected liver, lung, kidney, spleen, and blood samples 24 h post-infection. Whole tissues were homogenized in PBS supplemented with 0.1% Nonidet P-40. Serial dilutions of the homogenates were then plated on LB agar for colony counting (16).

### Serum killing assay

Bacterial susceptibility to normal human serum (NHS) was determined using a previously described method (17) with minor modifications. Human pooled serum was taken from three to five healthy volunteers. Bacterial cultures were incubated with NHS at two different concentrations (25% and 50%) for 1 and 3 h. Survival rates were determined by comparing the number of bacteria that remained viable after NHS treatment to the initial bacterial count before treatment. Experiments were carried out in triplicate, and the results were expressed as survival percentages.

### Transmission electron microscope observation

Transmission electron microscope (TEM) was employed to observe bacterial capsule structure. The procedure followed an adapted protocol from previous studies (18). In brief, for the bacteria capsule observation, 20 µL of freshly prepared 1% bovine serum albumin was applied to a carbon-coated copper grid for 30 s. After removing the solution, 20 µL of an overnight bacterial culture was added and allowed to sit for another 30 s. The grid was then gently immersed in 1% (wt/vol) phosphotungstic acid for 30 s. Before imaging, it was washed twice with PBS.

### Capsule uronic acid quantification assay

The extraction and quantification of uronic acid were performed to validate the capsule observation results and further quantify capsule production as previously described (19). The glucuronic acid content was determined from the standard curve and expressed as nanograms per 10^6^ CFU.

### Tellurite resistance test

MacConkey-potassium tellurite (MCKT) medium was prepared by following the manufacturer’s instructions for BD Difco MacConkey medium powder and supplementing it with varying amounts of potassium tellurite (Sigma-Aldrich, MA, United States) to achieve final potassium tellurite concentrations of 1, 2, 4, 8, 16, 32, 64, 128, 256, and 512 μg/mL. The potassium tellurite minimum inhibitory concentration (MIC) of *K. pneumoniae* was determined as the lowest concentration that inhibited bacterial growth (20).

### Antibiotic susceptibility testing

Antibiotic susceptibility was determined by disk diffusion assay against 15 antibiotics (amikacin [AN, 30 μg], ampicillin/sulbactam [SAM, 20 μg], cefazolin [CZ, 30 μg], cefepime [FEP, 30 μg], cefoxitin [FOX, 30 μg], ceftazidime [CAZ, 30 μg], ciprofloxacin [CIP, 5 μg], colistin [CL, 10 μg], ertapenem [ETP, 10 μg], imipenem [IPM, 10 μg], meropenem [MEM, 10 μg], levofloxacin [LVX, 5 μg], piperacillin/tazobactam [TZP, 100/10 μg], sulfamethoxazole/trimethoprim [SXT, 23.75/1.25 μg], and tigecycline [TIG, 15 μg]) (BD BBL Sensi-Disc, Becton, Dickinson and Company, MD, United States) following the Clinical and Laboratory Standards Institute (CLSI) guidelines (21). *E. coli* ATCC 25922 was used as a quality control strain. The results were interpreted according to the CLSI guidelines (21), except for tigecycline and colistin, which were interpreted according to the European Committee on Antimicrobial Susceptibility Testing standard (EUCAST, 2022) and based on a previous study (22), respectively. Isolates were classified as multidrug-resistant (MDR, resistance to at least one agent in three or more antimicrobial categories), extensively drug-resistant (XDR, resistance to all but one or two antimicrobial categories), and pandrug-resistant (PDR, resistance to all agents in all antimicrobial categories) based on criteria from a previous study (23).

### Statistical analysis

Statistical analyses were performed using GraphPad Prism software (version 10.4.0, United States). The mean and standard error of the mean were used to express all the results. Tukey’s method with one-way analysis of variance (ANOVA) was utilized to compare the differences between groups. A log-rank (Mantel-Cox) test was performed to compare the survival of larvae. A *P*-value *<* 0.05 was considered a significant difference.

## RESULTS

### Multiplex PCR assay for detecting the prevalence of five hvKp-associated biomarkers

To assess whether five genetic biomarkers, *peg-344*, *iroB*, *iucA*, *_p_rmpA*, and *_p_rmpA2*, combined with the phenotypic string test, can differentiate CRhvKp from carbapenem-resistant classical *K. pneumoniae* (CRcKp), we developed a multiplex PCR assay to detect these five biomarkers in 660 CRKp isolates (509 from Northern Taiwan and 151 from Southern Taiwan) and 69 LAKp isolates (29 from 2007 and 40 from 2017) (Table 1). These 69 LAKp isolates were all carbapenem-susceptible, with only two isolates classified as MDR (detailed antibiotic susceptibility to 15 antibiotics is shown in Table S2). Five CRKp isolates with different combinations of genetic biomarkers and two isolates (isolates CRKp35 and CRKp49) lacking all five biomarkers, confirmed by whole-genome sequencing, were used to evaluate the specificity and sensitivity of the multiplex PCR (Fig. S1). The results demonstrated high specificity, with no signals detected in isolates CRKp35 and CRKp49 (Fig. S1).

**TABLE 1:** Distribution of hypervirulence-associated genes and string test results of *Klebsiella pneumoniae*^*a*^

CRKp group genetic biomarkers–string tests	Genotypes and phenotypes (*n*, %)					
	Genetic biomarkers	Northern-CRKp (*n* = 509)	Southern-CRKp (*n* = 151)	2007-LAKp (*n* = 29)	2017-LAKp (*n* = 40)	Total
5 genes–string test positive	*peg-344* **–** *iroB* **–** *iucA* **–** *_p_rmpA* **–** *_p_rmpA2*	9 (1.8)	2 (1.3)	12 (41.4)	22 (55.0)	45
5 genes–string test negative	*peg-344* **–** *iroB* **–** *iucA* **–** *_p_rmpA* **–** *_p_rmpA2*	18 (3.5)	10 (6.6)	14 (48.3)	13 (32.5)	55
4 genes–string test negative	*peg-344* **–** *iucA* **–** *_p_rmpA* **–** *_p_rmpA2*	1 (0.2)	0	0	0	1
3 genes–string test positive	*peg-344* **–** *iroB* **–** *iucA*	1 (0.2)	0	0	1 (2.5)	2
3 genes–string test positive	*peg-344* **–** *iucA* **–** *_p_rmpA*	1 (0.2)	0	0	0	1
3 genes–string test negative	*peg-344* **–** *iroB* **–** *_p_rmpA*	0	0	0	1 (2.5)	1
2 genes–string test negative	*iucA* **–** *_p_rmpA2*	1 (0.2)	0	0	0	1
2 genes–string test negative	*peg-344* **–** *iroB*	0	0	2 (6.9)	1 (2.5)	3
1 gene–string test negative	*iucA*	1 (0.2)	0	0	0	1
0 gene–string test positive		2 (0.4)	1 (0.7)	1 (3.4)	1 (2.5)	5
Total		34 (6.7)	13 (8.6)	29 (100)	39 (97.5)	115

^*a*^ CRKp, carbapenem-resistant *Klebsiella pneumoniae*; LAKp, liver abscess *Klebsiella pneumoniae.*

Among the 69 LAKp isolates from the same hospital, 34 (49.3%) were positive for all genetic markers and the string test (defined as 5 genes-string test positive group), 27 (39.1%) were positive for all genetic markers but negative for the string test (defined as 5 genes-string test negative group), one isolate (1.4%) carried the *peg-344*, *iroB*, and *iucA* virulence genes and was string test positive, one isolate carried *peg-344*, *iroB*, and *_p_rmpA* but was string test negative, three isolates carried *peg-344* and *iroB* but was string test negative, and two isolates were only string test positive (Table 1). The proportion of LAKp isolates classified as the 5 genes-string test positive group was slightly higher in 2017 than in 2007 (55.0% in 2017 and 41.4% in 2007).

To investigate potential regional differences in the prevalence of these virulence genes, we analyzed 509 CRKp isolates from Northern Taiwan and 151 CRKp isolates from Southern Taiwan. The results showed that 1.8% (9 isolates) of the Northern isolates and 1.3% (2 isolates) of the Southern isolates belonged to 5 genes-string test positive group. Additionally, 18 isolates (3.5%) from Northern Taiwan and 10 isolates (6.6%) from Southern Taiwan were 5 genes-string test negative isolates. Furthermore, among the isolates from Northern Taiwan, 7 isolates carried 1–4 genetic markers or were positive only for the string test. Interestingly, only one CRKp isolate from Southern Taiwan was string test positive (without five biomarkers). Overall, the prevalence rate of the five genetic markers was significantly lower in CRKp isolates compared to LAKp isolates. Among the 47 non-liver abscesses CRKp isolates showing at least one biomarker or string test positivity, 23 (49.0%) were from blood, 9 (19.1%) from sputum, 13 (27.7%) from urine, and 2 (4.3%) from bronchial washings (Table S3). Additionally, 18 isolates (38.3%) were classified as MDR, 27 isolates (57.4%) as XDR, and 2 isolates (4.3%) as PDR (Table S2).

We further analyzed the prevalence of 5 genetic markers and string tests in 1,815 CSKp isolates (665 blood isolates and 1,150 urine isolates) to examine the prevalence of the virulence biomarkers and the string test positivity rate (Table S4 and Fig. S2). A total of 130 (130/665, 19.5%) and 37 (37/1,150, 3.2%) isolates were 5 genes-string test positive, derived from blood and urine samples, respectively (Fig. S2A). Moreover, 94 blood isolates (94/665, 14.1%) and 106 urine isolates (106/1,150, 9.2%) were 5 genes-string test negative (Table S4 and Fig. S2B). The proportion of blood isolates carrying all five biomarkers, regardless of string test positivity, was significantly higher than that of urine isolates (*P* < 0.001). The lower prevalence of virulence genes and reduced string test positivity observed in blood isolates from 2019 to 2022 may be attributable to the smaller number of isolates collected during this period, compared to other timeframes, or to changes in the predominant circulating clones. (Table S4). Additionally, among the 65 urine isolates collected in 1999, only one isolate had five biomarkers and string test positive, a proportion significantly lower than that of the other four collection periods.

Capsular-type specific primers were used to identify the eight most common capsular types (K1, K2, K5, K20, K47, K54, K57, and K64) in 367 carbapenem-susceptible *K. pneumoniae* isolates carrying five biomarkers regardless of their string test results (Table S5). Notably, K54 was not detected in our collection. Overall, K1 was the most prevalent (80 isolates, 21.8%), followed by K2 (30 isolates, 8.2%) and K64 (25 isolates, 6.8%). However, 191 isolates (52.0%) did not belong to any of the eight detected capsular types. Among the 167 5 genes-string test positive isolates, K1 (31 isolates, 18.6%) and K2 (18 isolates, 10.8%) were the most common. For the 200 5 genes-string test negative isolates, K1 (49 isolates, 24.5%), K2 (12 isolates, 6.0%), and K64 (13 isolates, 6.5%) were the most frequently detected capsular types (Table S5).

Further analysis of the distribution of these virulence genes and hypermucoviscosity phenotypes in relation to antibiotic resistance among CSKp isolates revealed significant differences in resistance rates across different isolate groups, except for tigecycline (*P* = 0.081) and colistin (*P* = 0.327), which had low resistance rates (Table S6). Overall, the 5 genes-string test positive isolates exhibited the lowest antibiotic resistance (except to colistin), followed by the 5 genes-string test negative isolates (except to amikacin, colistin, and tigecycline). Interestingly, the other groups of isolates (isolates that had at least one of the five genetic markers or were string test positive but did not include 5 genes-string test positive and 5 genes-string test negative isolates) showed the highest resistance, even surpassing cKp isolates in prevalence rates (Table S6).

### Evaluation of the virulence of different *K. pneumoniae* groups using a larvae infection model

**Fig 1:**
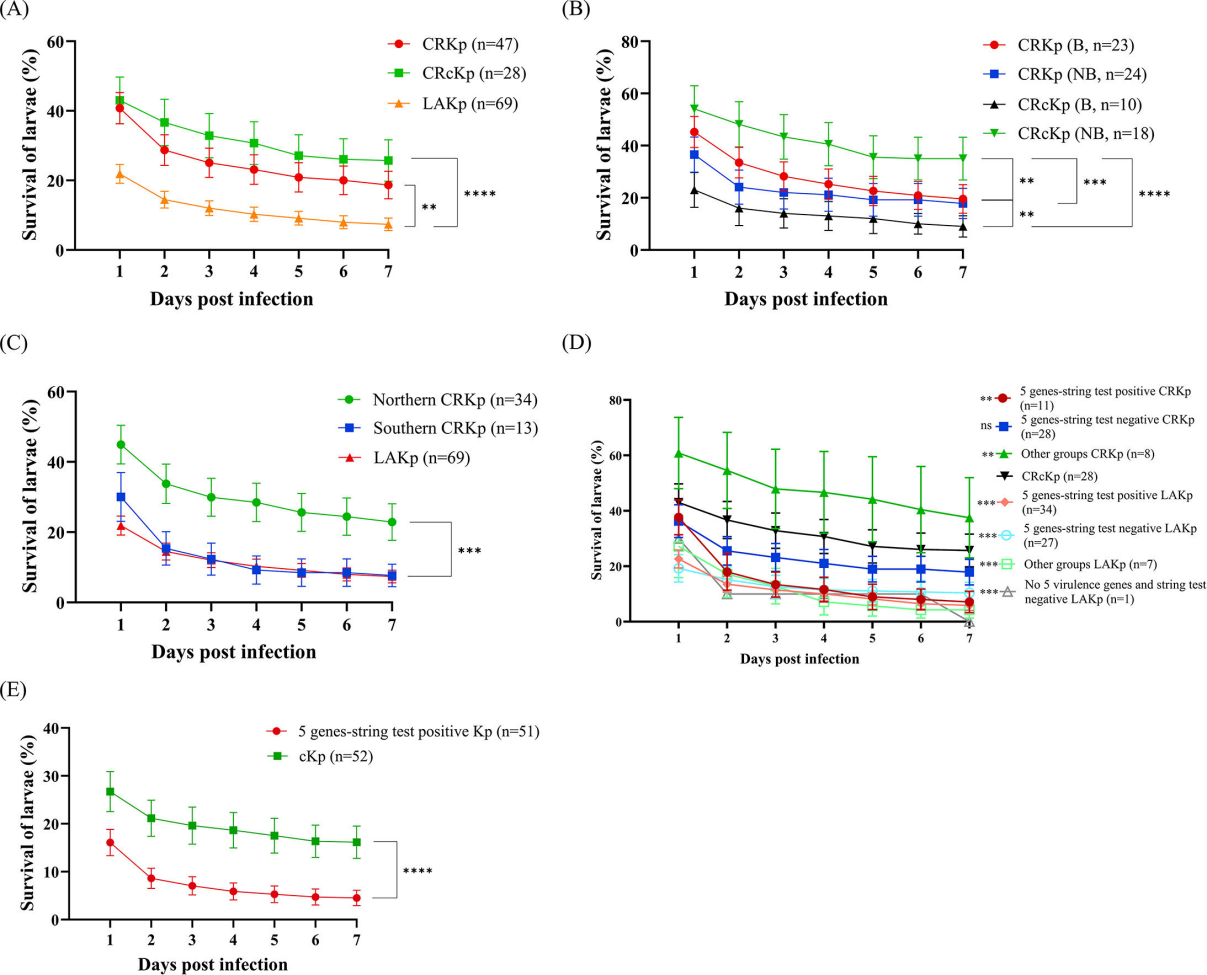
Virulence evaluation of *Klebsiella pneumoniae* using the *Galleria mellonella* infection model. (**A**) Survival percentages of larvae infected with CRKp, CRcKp, and LAKp over 7 days. (**B**) Comparison of larvae survival rates over 7 days when infected with strains isolated from blood (**B**) vs non-blood (NB) samples. (**C**) Larvae survival percentages following infection with CRKp isolates from Northern and Southern Taiwan. (**D**) Survival rates of larvae infected with various groups of CRKp and LAKp, including 5 genes-string test positive (positive for five biomarker genes and the string test), 5 genes-string test negative (positive for five biomarker genes but the string test negative), other groups, and CRcKp, over seven days. Statistical analyses were performed by comparing each group with the CRcKp group. (**E**) Evaluation of the virulence of 5 genes-string test positive carbapenem-susceptible *K. pneumoniae* and classical *K. pneumoniae* (randomly selected from 1999, 2004, 2009, 2014, and 2019–2022). To evaluate differences in larval survival across experimental groups, survival curves were compared using the log-rank (Mantel–Cox) test. Statistical significance is indicated as follows: **, *P* < 0.01; ***, *P* < 0.001; ****, *P* < 0.0001; ns, not significant. CRKp, carbapenem-resistant *K. pneumoniae*; CRcKp, carbapenem-resistant classical *K. pneumoniae*; LAKp, liver abscess *K. pneumoniae*.

We randomly selected 28 CRcKp isolates that lacked all five virulence genes and tested negative for string tests as the control group. The virulence of the CRKp groups listed in Table 1 was compared against these 28 CRcKp isolates and 69 LAKp isolates using the larvae infection model (Fig. 1). LAKp showed the highest virulence to larvae, with only 7.4% of larvae surviving 7 days post-infection, followed by CRKp isolates carrying at least one of the five target genetic markers or testing positive for the string test (18.7% survival rate). CRcKp isolates exhibited relatively low virulence to larvae, with a larvae survival rate of 25.7% 7 days post-infection (Fig. 1A).

Among the 47 CRKp isolates carrying at least one virulence biomarker or testing positive in the string test, 23 putative hypervirulent CRKp isolates were obtained from blood, 13 from urine, 9 from sputum, and 2 from bronchial washings (Table S3). We aimed to determine whether the source of bacterial isolates is associated with the virulence of these isolates in larvae. Our findings revealed no significant differences in larvae survival rates 7 days post-infection among putative hypervirulent CRKp isolates from blood (19.6%) compared to non-blood isolates (17.8%) (Fig. 1B). However, these CRKp isolates, regardless of their source, exhibited higher virulence against larvae compared to non-blood CRcKp isolates (35.0% survival rate 7 days post-infection). Additionally, blood-isolated CRcKp demonstrated lower larvae survival rates 7 days post-infection (9.0%) compared to non-blood CRcKp isolates (Fig. 1B).

Additionally, among the 47 CRKp isolates carrying at least one virulence biomarker or testing positive in the string test, 34 isolates were collected from hospitals in Northern Taiwan, while 13 were from Southern Taiwan (Table 1). Notably, isolates from Southern Taiwan exhibited a larval average survival rate of 7.7% 7 days post-infection and carried five virulence genes (except one isolate was only string test positive) (Fig. 1C). This indicates that their virulence toward larvae is relatively higher compared to isolates from Northern Taiwan, which showed a survival rate of 22.9%. Furthermore, the virulence of the Southern Taiwan isolates was comparable to that of LAKp strains (7.4% survival rate), likely because most of these isolates had at least five virulence genes (Fig. 1C).

We further assessed the virulence of isolates from different groups classified in Table 1 based on various combinations of virulence biomarkers and string test results. Overall, the 7-day post-infection survival rates of larvae infected with 5 genes-string test positive, 5 genes-string test negative, and other groups LAKp isolates were 5.9%, 10.4%, and 4.3%, respectively (Fig. 1D). For CRKp isolates, the corresponding survival rates were 7.1%, 17.9%, and 37.5% after 7 days. Additionally, larvae infected with CRcKp isolates exhibited a survival rate of 25.7% at the 7-day post-infection. Notably, larvae infected with other groups CRKp and CRcKp isolates displayed relatively higher survival rates after 1 day, at 60.8% and 43.0%, respectively. These findings indicate that LAKp isolates exhibit consistently high virulence regardless of their virulence factor profiles or string test results. In contrast, among CRKp isolates, those carrying all five genetic markers demonstrated higher virulence, especially those that were also string test-positive, exhibiting virulence levels comparable to LAKp (Fig. 1D).

Five genes-positive CRKp isolates demonstrated significantly higher virulence compared to CRcKp. To further validate whether the five biomarker genes and hypermucoviscosity contribute to the virulence of *K. pneumoniae* regardless of carbapenem susceptibility, we randomly selected 10–12 cKp and 5 genes-string test positive CSKp isolates from each period, 1999, 2004, 2009, 2014, and 2019–2022, among the 1,815 CSKp shown in Table S4. The results indicated that 51 5 genes-string test positive *K. pneumoniae* isolates exhibited hypervirulence in the larvae model, with a 4.5% survival rate, compared to 52 cKp isolates, which had a 16.2% survival rate, after 7 days of infection (Fig. 1E).

### Validation of hypervirulent *K. pneumoniae* using a mouse bacteremia model

**Fig 2:**
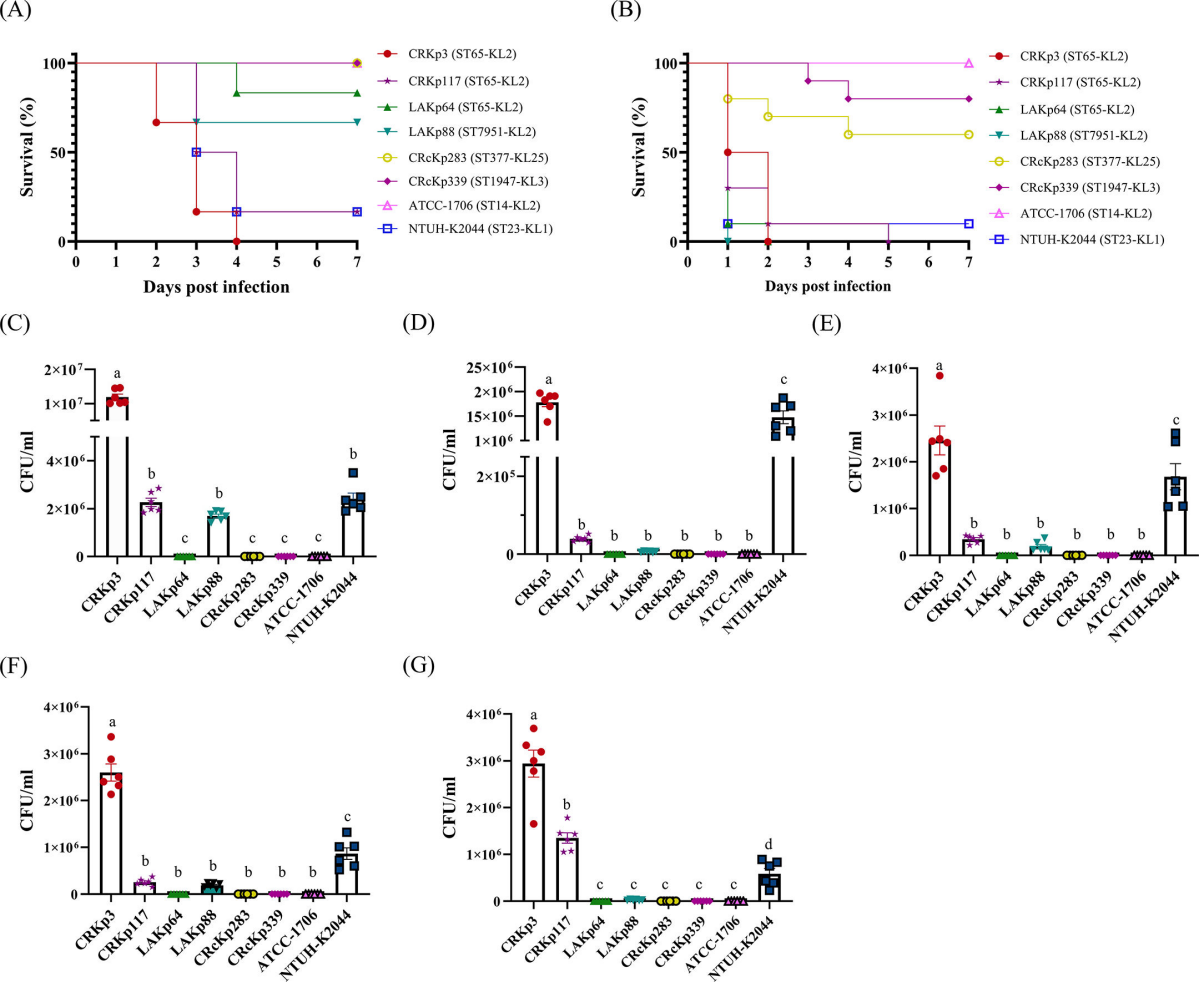
Evaluation of bacterial virulence using mouse and larvae infection models. (**A**) Assessment of the virulence of CRKp3, CRKp117, LAKp64, LAKp88, CRcKp283, and CRcKp339 using a mouse bacteremia model. NTUH-K2044 and ATCC-1706 served as positive and negative controls, respectively. (**B**) Survival rates of larvae 7 days post-infection with the tested strains. Bacterial burdens in mice infected with each strain were measured in various organs: liver (**C**), lung (**D**), spleen (**E**), kidney (**F**), and blood (**G**). Differences among groups were analyzed using one-way ANOVA with Tukey’s multiple comparisons test. Different letters indicate statistically significant differences (*P* < 0.05).

To further validate the larvae infection results, we employed a mouse bacteremia infection model. We selected CRKp3 and CRKp117, both ST65-K2 isolates carrying all five virulence markers and string test positive, as well as LAKp64, an ST65-K2 isolate with similar characteristics, and LAKp88, an ST7951-K2 strain carrying all five virulence markers but string test negative. NTUH-K2044, a ST23-K1 strain previously recognized as hypervirulent (24), was used as the positive control. However, since no ST65 or ST7951 were identified among our CRcKp isolates, we selected CRcKp339 (ST1947-K3), CRcKp283 (ST377-KL125), and ATCC BAA-1706 (ST14-K2) to validate their lower virulence in mice (Fig. 2). In our previous study, ATCC BAA-1706 was shown to exhibit low virulence in larvae (14), lacking all five key virulence biomarkers and showing a negative result in the string test. Therefore, it served as a low-virulence control strain in these mouse infection experiments. The results showed that infection with NTUH-K2044 resulted in five of six mice died within 7 days (16.7% survival rate), which was similar to the virulence of CRKp117. Surprisingly, all mice infected with CRKp3 died within 4 days. Interestingly, LAKp64 and LAKp88 caused only one and two mice deaths, respectively, among the infected mice over 7 days. In contrast, all mice infected with CRcKp339, CRcKp283, or ATCC BAA-1706 survived (Fig. 2A). The larvae infection model also revealed that the high 7-day survival rate for larvae infected with CRcKp339 (80%), CRcKp283 (60%), and ATCC BAA-1706 (100%) (Fig. 2B). In contrast, all larvae infected with CRKp3, LAKp64, and LAKp88 died within 2 days, while those infected with CRKp117 all died within 5 days. In addition, larvae infected with NTUH-K2044 showed only 10% survival at 7 days post-infection.

We further analyzed the bacterial loads in the liver (Fig. 2C), lung (Fig. 2D), spleen (Fig. 2E), kidney (Fig. 2F), and blood (Fig. 2G) 24 h post-infection. Overall, the highest bacterial loads were observed in mice infected with CRKp3, which also exhibited the highest mortality (Fig. 2A), followed by NTUH-K2044. In the liver, mice infected with CRKp3, CRKp117, LAKp88, and NTUH-K2044 showed bacterial loads of approximately 1.0 × 10^7^, 2.3 × 10^6^, 1.7 × 10^6^, and 2.4 × 10^6^ CFU, respectively (Fig. 2C). In the lung, CRKp3 and NTUH-K2044 caused a bacterial load of 1.8 × 10^6^ and 1.5 × 10^6^ CFU, respectively, whereas CRKp117 exhibited a lower load of approximately 4 × 10^4^ CFU (Fig. 2D). In the spleen, CRKp3 and NTUH-K2044 reached 2.5 × 10^6^ and 1.7 × 10^6^ CFU, respectively, while CRKp117 and CRKp88 had a lower count of 3.5 ×10^5^ and 1.9 × 10^5^ CFU, respectively (Fig. 2E). In the kidney, CRKp3, NTUH-K2044, CRKp117, and LAKp88 showed approximately 2.6 × 10^6^, 8.6 × 10^5^, 2.6 × 10^5^, and 1.9 × 10^5^ CFU, respectively (Fig. 2E). In the blood, the bacterial load for CRKp3 (2.9 × 10^6^ CFU) was higher than CRKp117 (1.4 × 10^6^ CFU) and NTUH-K2044 (5.9 × 10^5^ CFU). In contrast, no significant bacterial presence was detected in the liver, lung, spleen, kidney, or blood of mice infected with CRcKp283, CRcKp339, ATCC BAA-1706, and LAKp64 (Fig. 2C through G).

### Hypervirulent *K. pneumoniae* in general exhibited a thicker capsule and higher tellurite resistance phenotypes

To characterize phenotypic traits associated with the hypervirulence of CRKp, we evaluated serum resistance, capsular polysaccharide production, biofilm formation, iron acquisition, and tellurite resistance across different isolate groups shown in Table 1. The phenotype results of individual isolates are detailed in Table S7.

**Fig 3:**
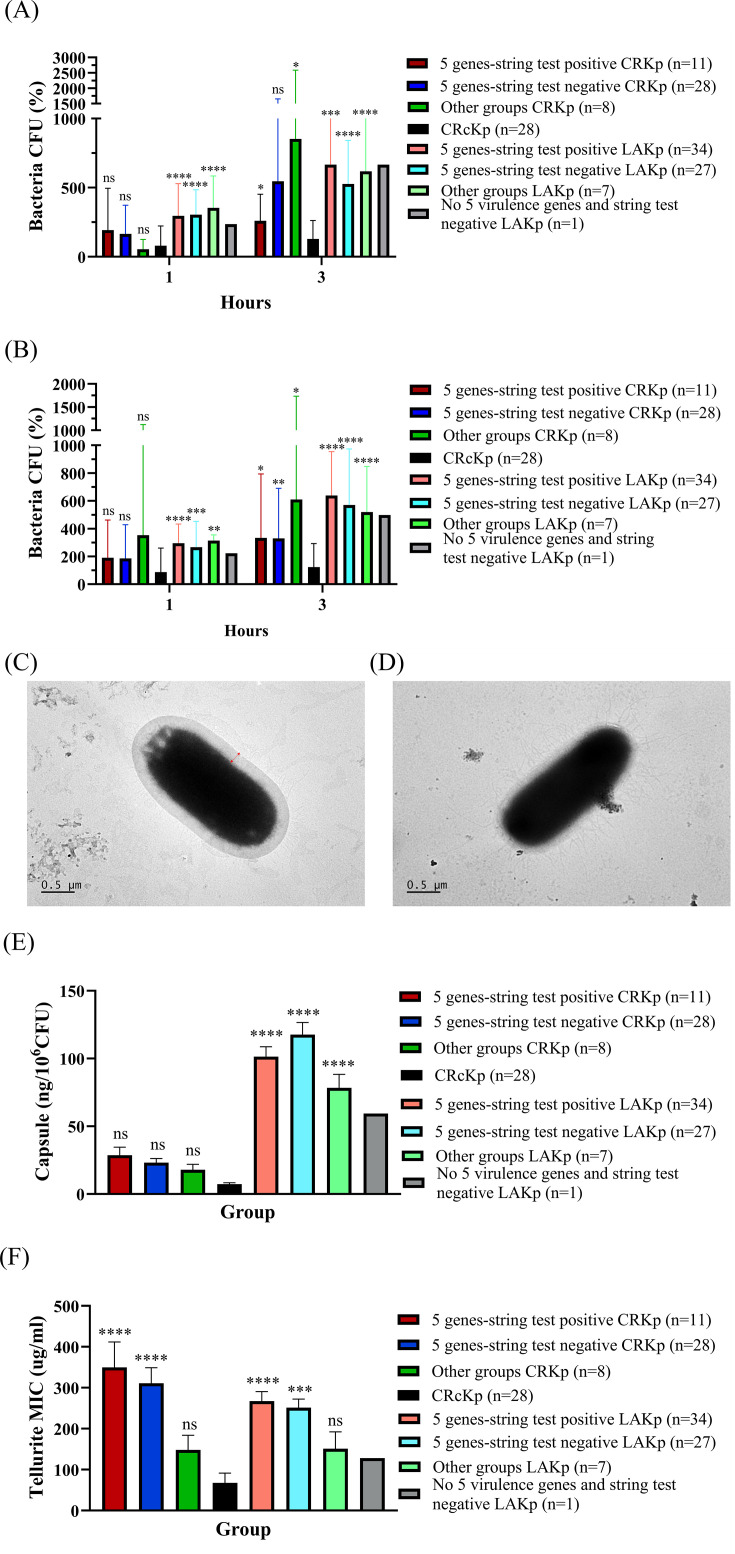
Serum resistance, capsule production, and tellurite minimum inhibitory concentration in CRKp, CRcKp, and LAKp. Resistance of *K. pneumoniae* isolates to 25% (**A**) and 50% (**B**) pooled human serum after 1-h and 3-h co-incubation. TEM images showing the capsule of CRKp3 (**C**) and CRcKp ATCC BAA-1706 (**D**), with bidirectional red arrows indicating the thickness of the bacterial capsule. (**E**) Capsule quantification through uronic acid detection in CRKp, CRcKp, and LAKp. (**F**) Tellurite MIC of five different groups. One isolate CRKp1256 with MIC >512 µg/mL, therefore, was calculated as 1,024 for this assay. Statistical analysis was performed to compare each isolate group with the CRcKp group. LAKp without the five biomarkers and string test-negative had only one isolate; therefore, statistical analysis was not performed. Differences among groups were analyzed using one-way ANOVA with Tukey’s multiple comparisons test. Statistical significance is indicated as follows: *, *P* < 0.05; **, *P* < 0.01; ***, *P* < 0.001; ****, *P* < 0.0001; ns, not significant.

After 1 h of exposure to 25% serum, *K. pneumoniae* isolates from the 5 genes-string test positive, 5 genes-string test negative, and other LAKp groups showed 2.96-, 3.04-, and 3.55-fold increases in bacterial counts, respectively, while CRcKp and other groups CRKp exhibited only 54.3% and 80.3% survival, respectively (Fig. 3A). In contrast, 5 genes-string test positive and 5 genes-string test negative CRKp showed modest increases of 1.93- and 1.65-fold, respectively. After 3 h, other groups CRKp increased by 8.52-fold, while 5 genes-string test positive, 5 genes-string test negative, and other groups LAKp exhibited 6.66-, 5.27-, and 6.18-fold increases, respectively; CRcKp showed only a 1.29-fold increase. Moreover, 5 genes-string test positive and 5 genes-string test negative CRKp showed a 2.59- and 5.46-fold increase in bacterial growth.

Under 50% serum conditions, similar trends were observed (Fig. 3B). After 1 h, 5 genes-string test positive, 5 genes-string test negative, and other groups LAKp increased by 2.95-, 2.66-, and 3.14-fold, while CRcKp maintained 87.7% survival. Moreover, 5 genes-string test positive, 5 genes-string test negative, and other groups CRKp showed a 1.90-, 1.86-, 3.52-fold increase in bacterial growth, respectively. After 3 h, other groups CRKp reached a 6.10-fold increase, while 5 genes-string test positive, 5 genes-string test negative, and other LAKp groups increased by 6.38-, 5.70-, and 5.19-fold, respectively; CRcKp increased only 1.23-fold. Notably, the 5 genes-string test positive and 5 genes-string test negative CRKp groups reached 3.33- and 3.30-fold increases, respectively.

Capsule structure and quantification were analyzed for CRKp isolates, CRcKp, and LAKp isolates to evaluate the relationship between capsule production and bacterial virulence (Fig. 3C through E). TEM revealed a large, clear capsule zone surrounding isolate CRKp3 (Fig. 3C). In contrast, no obvious capsule-surrounded CRcKp ATCC BAA-1706 isolate was observed (Fig. 3D). LAKp exhibited the thickest average capsule, followed by 5 genes-string test positive CRKp, 5 genes-string test negative CRKp, other groups CRKp, and CRcKp3. No significant differences in capsule thickness were detected between CRcKp and any groups of CRKp (data not shown). These findings were supported by the uronic acid quantification assay, which showed that 5 genes-string test negative LAKp produced the highest amount of capsule (117.7 ng/10^6^ CFU), followed by 5 genes-string test positive LAKp (101.4 ng/10^6^ CFU), other groups LAKp (78.5 ng/10^6^ CFU), 5 genes-string test positive CRKp (28.7 ng/10^6^ CFU), 5 genes-string test negative CRKp (23.1 ng/10^6^ CFU), other groups CRKp (18.0 ng/10^6^ CFU), and CRcKp (7.4 ng/10^6^ CFU) (Fig. 3E).

Tellurite resistance with high MIC is also considered a phenotypic method for distinguishing hvKp from cKp. Our results showed that the average tellurite MIC for CRcKp was 67.8 µg/mL, significantly lower than that of the 5 genes-string test positive CRKp (350.0 µg/mL, *P* < 0.0001), 5 genes-string test negative CRKp (310.9 µg/mL, *P* < 0.0001), 5 genes-string test positive LAKp (267.3 µg/mL, *P* < 0.0001), and 5 genes-string test negative LAKp (251.3 µg/mL, *P* < 0.001). However, the tellurite MICs of other groups CRKp and LAKp were only 148.3 µg/mL and 151.1 µg/mL, respectively, showing no statistically significant difference compared with CRcKp (Fig. 3F).

Compared to cKp, hvKp typically harbors additional siderophore-encoding genes, such as *iroB* and *iucA*, which enhance iron acquisition capacity. To assess this phenotype, a CAS agar assay was employed to evaluate siderophore-mediated iron acquisition. The results revealed that 5 genes-string test positive LAKp exhibited higher iron acquisition ability (5.7 mm) than 5 genes-string test negative LAKp (5.2 mm), other groups CRKp (3.8 mm), other groups LAKp (3.2 mm), 5 genes-string test negative CRKp (3.0 mm), CRcKp (3.0 mm), and 5 genes-string test positive CRKp (2.6 mm) after 24 h (Fig. S3A). After 48 h of incubation, the 5 genes-string test positive LAKp displayed the strongest siderophore activity (12.4 mm), followed by 5 genes-string test negative LAKp (11.7 mm), other groups CRKp (7.4 mm), other groups LAKP (7.1 mm), 5 genes-string test negative CRKp (6.2 mm), 5 genes-string test positive CRKp (5.2 mm), and CRcKp (4.7 mm) (Fig. S3B).

Previous studies indicated that hvKp produces more biofilm than cKp (25). Therefore, we utilized a microtiter plate assay combined with crystal violet staining to assess the biofilm formation of the tested isolates (Fig. S4). In nutrient-rich LB, 5 genes-string test negative LAKp isolates formed the highest amount of biofilm on the first and second days of incubation, while on the third day, other groups LAKp isolates exhibited the highest biofilm formation. Overall, LAKp isolates produced more biofilm than CRKp and CRcKp (Fig. S4A). In nutrient-limited M9 medium, the biofilm-forming ability of CRcKp significantly increased, reaching levels comparable to LAKp isolates. Similarly, for CRKp, apart from other groups CRKp, which formed a higher amount of biofilm, the biofilm formation ability of 5 genes-string test positive and 5 genes-string test negative CRKp remained low similar to their biofilm formation in LB (Fig. S4B). Therefore, in *in vitro* assays used to screen for hvKp, in addition to the string test, capsule hyperproduction and high tellurite MIC were also positively associated with hypervirulent isolates.

### MLST analysis of CRKp and LAKp

**Fig 4:**
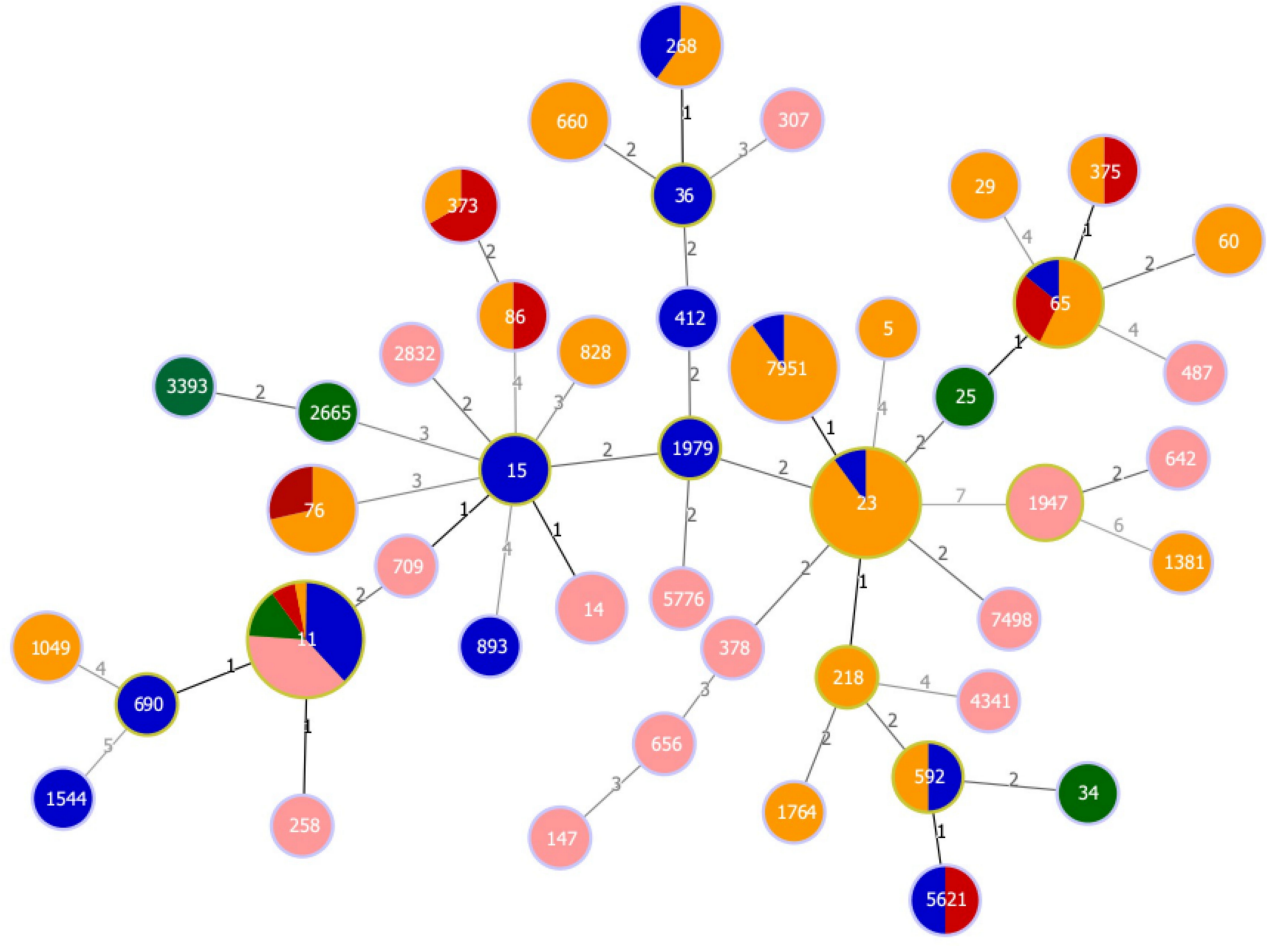
Minimum-spanning tree obtained with PHYLOViZ for the MLST profiles of 47 CRKp, 28 CRcKp, and 69 LAKp isolates. Numbers on branches represent the number of loci different from that of the founder ST. The red color indicates isolates that possess 5 genes and are string test positive, the deep blue color indicates isolates that possess 5 genes but are string test negative, the green color represents other CRKp isolates, the orange-yellow color represents LAKp isolates, and the pink color represents CRcKp isolates. The size of each MLST node represents the number of isolates.

MLST analysis of 47 CRKp and 69 LAKp isolates revealed a high level of sequence type diversity, encompassing 45 distinct STs (Fig. 4). Among the 11 5 genes-string test positive isolates and 28 5 genes-string test negative isolates, the most prevalent sequence types were ST11 (*n* = 13, 33.3%), ST65 (*n* = 3, 7.7%), and ST268 (*n* = 3, 7.7%). In the 69 LAKp isolates, ST23 and ST7951 were the most common, each accounting for 18 isolates (26.1%), followed by ST76 (*n* = 5, 7.2%), ST65 (*n* = 4, 5.8%), and ST660 (*n* = 4, 5.8%). For CRcKp, ST11 was the most frequent (*n* = 11, 39.3%), followed by ST1947 (*n* = 3, 4.3%) (Fig. 4). Notably, ST7951 was reported for the first time.

## DISCUSSION

Since the 1990s, pyogenic liver abscesses caused by hvKp strains have become a major epidemiologic problem in Southeast Asia and now represent >80% of pyogenic liver abscesses in Asia (2, 3). Detection methods for hvKp have been the subject of controversy despite their characterization for more than 30 years (26). Clinical symptoms and positive string tests are commonly used indicators of hvKp identification (27). However, distinguishing hvKp from cKp is increasingly challenging due to overlapping clinical symptoms and the occurrence of healthcare-associated infections (28). The presence of the hypermucoviscosity phenotype in cKp further complicates the use of string tests for hvKp identification (10). Recent studies have highlighted the effectiveness of specific biomarkers, such as *iroB*, *iucA*, *_p_rmpA*, *_p_rmpA2*, and *peg-344*, in accurately identifying hvKp isolates and assessing their survival in animal models (10). A study conducted in a hospital in China from 2008 to 2012 reported a detection rate of 31.4% (22/70) based on positive string tests (29). Another study in a North Chinese hospital found that 52.2% (59/113) of *K. pneumoniae* isolates were positive for *_p_rmpA*, *_p_rmpA2*, *iucA*, *iroB*, *peg-344*, and *peg-589*, along with a positive string test (26). In a nationwide study conducted in China, the prevalence of aerobactin-positive hvKp isolates varied between cities, with rates ranging from 8.3% to 73.9% (30). These results suggest that the combination of biomarker detection and phenotypic string test could be used to accurately detect hvKp.

The differences in the criteria used to define hvKp in previous studies may contribute to variations in reported prevalence. In this study, strains lacking all five hvKp-associated biomarker genes did not exhibit hypervirulence in larvae. Our data revealed that 5 genes-string test positive *K. pneumoniae* isolates showed a similar virulence level to larvae, compared to the LAKp, regardless of their carbapenem susceptibility. Furthermore, larvae infected with 5 genes-string test positive CRKp were more virulent than the 5 genes-string test negative CRKp, with survival rates of 5.9% and 17.9%, respectively. These results indicate the contribution of hypermucoviscosity phenotype to *K. pneumoniae* virulence. In addition, a strain carrying at least five virulence genes (*peg-344*, *iroB*, *iucA*, *_p_rmpA*, and *_p_rmpA2*) simultaneously could be preliminarily classified as hvKp. Moreover, based on previous literature reporting that the string test should not be used as a definitive diagnostic test for hvKp, particularly in low-prevalence areas, where its performance characteristics will lead to substantially more erroneous classifications than with the other more accurate markers identified (10), we suggest the string test is helpful as an additional assay for assessing bacterial virulence. Specifically, isolates positive in the string test exhibit stronger virulence in larvae compared with isolates carrying all five virulence genes. Therefore, the use of these five biomarkers and/or with the string test in future studies could facilitate more accurate detection of hvKp prevalence across different regions and enable further characterization of strain-specific features.

In recent years, loop-mediated isothermal amplification (LAMP) has been applied for rapid detection of hvKp. For example, the Eazyplex hvKp LAMP assay can simultaneously detect six virulence-associated genes (*iucC*, *iroC*, *rmpA*, *rmpA2*, *ybt*, and *clb*) and provide a Kleborate score, allowing rapid assessment of bacterial virulence and providing valuable information for clinical decision-making (31, 32). Therefore, in future clinical applications, if a single colony of *K. pneumoniae* is cultured from clinical specimens, a LAMP assay targeting these five genes combined with a string test could allow determination of whether the patient is infected with hvKp within approximately 1 h. This workflow is not only cost-effective but also time-efficient, making it feasible for clinical implementation. However, the selection of target virulence genes remains critical to maximize the specificity and accuracy of hvKp identification.

Although the *G. mellonella* larvae infection model may not always show results consistent with mouse infection models, its high-throughput capability and low cost make it a widely used alternative for assessing bacterial virulence (33–35). In this study, CRKp3, CRKp117, and LAKp64 belong to ST65-K2 isolates and carry all five virulence markers, showing positive string test results. In the *G. mellonella* infection model, all three isolates caused complete larvae mortality within 2–5 days. However, in the mouse infection model, CRKp3 killed all infected mice within 4 days, CRKp117 caused 5 out of 6 mice deaths by day 7, whereas LAKp64 resulted in the death of only one mouse within 7 days. Notably, different models possess distinct immune systems. The larval immune system of *G. mellonella* shows remarkable structural and functional similarities to the innate immune response of mammals, but no adaptive immune system has been reported (36). Consequently, variations in immune mechanisms among hosts can lead to model-dependent differences in virulence outcomes. Furthermore, the virulence factors required for infection vary across hosts. In *G. mellonella* larvae, the predominant role of surface polysaccharides in infection, along with additional contributions from siderophores, cell envelope proteins, purine biosynthesis genes, and several genes with unknown functions (37). However, this invertebrate model lacks the vertebrate-specific adaptive immune response and, thus, cannot fully represent the complexity of mammalian host–pathogen interactions (35). In contrast, murine infection models have demonstrated that capsule type (particularly K1 and K2), siderophore systems (such as aerobactin and yersiniabactin), and fimbrial adhesins (FimK) are key determinants of pneumonia and systemic infections (38, 39). However, these results emphasize the complementary nature of these two models: *G. mellonella* larvae enable high-throughput screening of virulence-associated genes, whereas murine models better recapitulate clinical infection processes and immune responses. Finally, variations in virulence factor expression levels and the presence of strain-specific single nucleotide polymorphisms further complicate the identification of universal virulence indicators. Therefore, virulence was interpreted at the population level in this study, and model-specific optimization remains necessary to define the most suitable indicators.

Previous studies have suggested that capsule production may serve as one of the indicators for bacterial virulence, and our data also support a correlation between capsule abundance and virulence in the larvae infection model. Interestingly, the CRKp groups, including both 5 genes-string test positive and 5 genes-string test negative CRKp isolates, exhibited higher capsule production compared to other CRKp groups and CRcKp. However, the difference was not statistically significant. Meanwhile, the LAKp groups displayed substantial capsule production. These findings suggest that isolates harboring all five hypervirulence genes tend to produce thicker capsules compared to *K. pneumoniae* groups lacking one or more of these genes. The thickness of the capsule correlated with larval survival rates, as isolates with thinner capsules demonstrated higher survival rates. Consistently, previous research has shown that capsule size is a strong predictor of bacterial pathogenicity (40). However, in the complex *in vivo* environment of the host, a single virulence contributor alone is insufficient to establish infection. Therefore, while capsule production remains a hallmark of hypervirulence, it should be interpreted as a contributing factor that acts together with other genetic and physiological traits to define the overall virulence phenotype of *K. pneumoniae*.

Serum-killing assays in our study further supported this complexity: isolates with thinner capsules were more susceptible to serum, yet certain non-hypercapsulated CRKp isolates still showed unexpectedly high serum resistance, suggesting additional mechanisms—potentially involving outer membrane proteins or surface polysaccharides—also contribute to immune evasion. Collectively, these results underscore that hypervirulence in *K. pneumoniae* arises from the combined effects of capsule regulation, iron acquisition, and plasmid-borne virulence genes rather than any single phenotypic trait.

Previous studies have demonstrated that tellurite resistance is strongly associated with hvKp clonal group 23 (CG23), CG65, and CG86, as the *terW* gene is often located on virulence plasmids (8, 9, 20, 41). However, our unpublished data revealed that the presence of *terW* did not always correlate with tellurite minimum inhibitory concentration levels, and certain virulence plasmids lacked *terW* entirely. Wu and colleagues reported that the *terW* gene was detected in 70.6% (60 of 85) of hvKp strains and 13.3% (15 of 113) of cKp strains (20). In a comprehensive *in silico* analysis of 79 hvKp plasmids, 66 (83.5%) were found to harbor the *terB* gene, indicating that while *terB* is prevalent, it is not universally present across all hypervirulent plasmids (42). These observations suggest that the presence of the *ter* operon alone is insufficient to predict hypervirulent potential. Although tellurite resistance may serve as a supportive screening indicator, suggesting the possible presence of virulence plasmids, it lacks the specificity required to be used as an independent diagnostic marker and is relatively time-consuming and complex. Therefore, combining genotypic analyses with phenotypic string test assays provides a more reliable and practical strategy for identifying hvKp in clinical laboratories.

Our study still has several limitations: (i) The number of CRKp isolates carrying virulence biomarkers or testing positive in the string test remains small. (ii) Although the combination of virulence biomarkers and the string test can reliably indicate bacterial virulence, the incorporation of additional phenotypes and genotypes to enhance the rapid and accurate identification of hvKp remains to be explored. (iii) The mere presence of virulence genes does not provide insight into their expression under different stress conditions in the host. This aligns with our observation that LAKp exhibits relatively lower virulence in the mouse model, possibly due to lower gene expression. Moreover, mutations in the promoters or open reading frames of these genes may impact their function and expression, necessitating further investigation. (iv) Additionally, factors such as the CFU used for infecting larvae and mice, as well as the specific strains tested, could influence the virulence results in the mouse model. While the larvae model may not fully correlate with the mouse model, it still holds value for screening highly virulent strains. (v) In this study, we identified a novel ST7951 and uncovered genomic and phenotypic differences between LAKp and CRhvKp. However, the characteristics of these unique clones require further exploration.

In conclusion, we found that the presence of five hypervirulence genes (*iucA*, *iroB*, *peg-344*, *_p_rmpA*, and *_p_rmpA2*), hypermucoviscosity, and thicker capsule, contribute together to hypervirulence in *K. pneumoniae*. The dissemination of hvKp strains represents a serious public health concern, warranting close monitoring of their spread and evolving characteristics.

## Data Availability

All data supporting the findings of this study are included within the paper and supplemental material.

## SUPPLEMENTAL MATERIAL

Supplemental figures spectrum.02474-25-s0001.doc Figures S1 to S4.

Supplemental tables spectrum.02474-25-s0002.xls Tables S1 to S7.

ASM does not own the copyrights to Supplemental Material that may be linked to, or accessed through, an article. The authors have granted ASM a non-exclusive, world-wide license to publish the Supplemental Material files. Please contact the corresponding author directly for reuse.
