# Supplementary material for: A combined genetic and phenotypic marker approach enables precise detection of hypervirulent Klebsiella pneumoniae and reveals associated traits of capsule overproduction and tellurite resistance
Source: Microbiol Spectr. 2026 Jan 7;14(2):e02474-25. doi: 10.1128/spectrum.02474-25 (PMC12889066; doi:10.1128/spectrum.02474-25)
Supplement: Supplemental figures — Figures S1 to S4. [file spectrum.02474-25-s0001.doc]

**Methods**

**Identification of virulence genes by using multiplex PCR**

Multiplex PCR was conducted on a GeneExplorer Thermal Cycler (Hangzhou Bioer Technology, China) using Taq 2x Master Mix (Ampliqon, Denmark). The reaction conditions included an annealing temperature of 59°C and 25 amplification cycles. PCR products were visualized on agarose gels stained with ethidium bromide and examined under ultraviolet light. The primer sequences and their expected product sizes are listed in **Table S1**.

**Chrome azurol S (CAS) assay**

Chrome azurol S (CAS) agar was prepared based on previously established protocol **(1)** with slight modifications. Bacteria were cultured on LB agar at 37°C overnight. Using a 10 μL pipette tip, single colonies were picked and inoculated onto CAS agar. The CAS agar plates were incubated at 37°C for 24 and 48 hours before measuring the diameter of the orange zone.

**Biofilm formation**

Biofilm assays were performed as described previously **(2)**, with slight modifications. Bacterial cultures were diluted to an OD_600_ of one and incubated in microtiter plates for 24 hours. After washing and fixing, crystal violet staining was done, followed by quantification at 595 nm.

**Figure S1.**


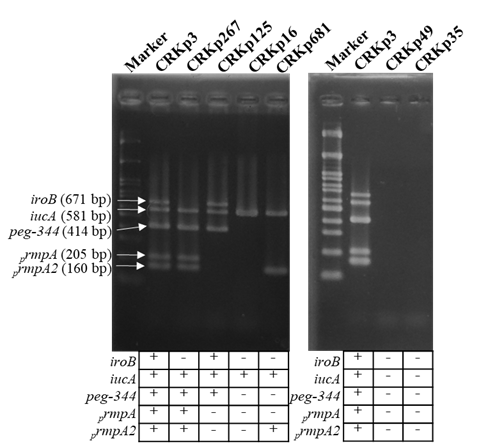


**Fig. S1.** **Establishment of multiplex PCR to detect hypervirulence-associated genes, including *iroB*, *iucA*, *peg-344*, *_p_rmpA*, and *_p_rmpA2***. Seven CRKp isolates (CRKp3, CRKp267, CRKp125, CRKp16, CRKp681, CRKp49, and CRKp35) with whole-genome sequencing results were used to validate the specificity of the multiplex PCR for detecting the five biomarkers. The whole-genome sequencing analysis results of each strain are shown below the agarose gel image.

**Figure S2.**


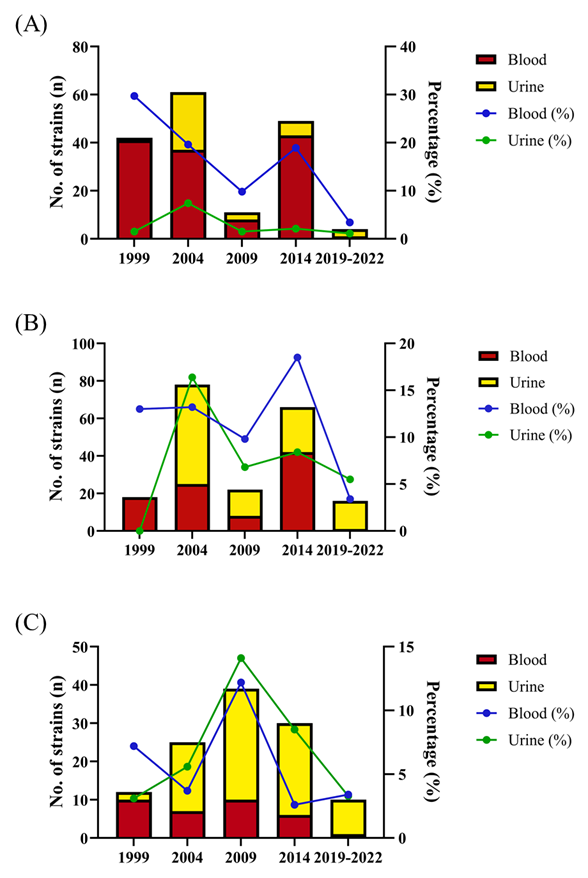


**Fig. S2. Distribution of virulence genes and string test results of carbapenem-susceptible *Klebsiella pneumoniae* from 1999 to 2022.** **(A).** Isolates have five virulence genes and testing positive in the string test. **(B).** Isolates have five virulence genes but testing negative in the string test. **(C).** Other groups refer to isolates with at least one of the five target virulence genes or tested positive in the string test, but do not belong to the 5 genes-string test positive or 5 genes-string test negative groups. The bar chart represents the number of isolates, while the line chart indicates the percentage.

**Figure S3**.


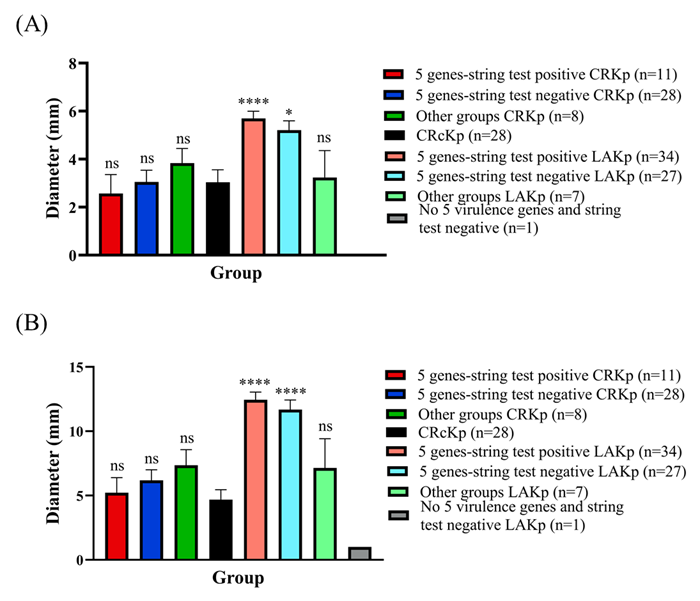


**Fig. S3**. **Quantitative iron acquisition ability of CRKp, CRcKp, and LAKp using the chrome azurol S (CAS) agar assay.** Quantitative measurements of iron acquisition ability were determined after 24 hours **(A)** and 48 hours **(B)** of incubation. The CRcKp group was used as the control group, and statistical analysis was conducted in comparison with this group. Differences among groups were analyzed using one-way ANOVA with Tukey’s multiple comparisons test. Statistical significance is indicated as follows: *, *p* < 0.05; ****, *p* < 0.0001; ns, not significant.

**Figure S4.**


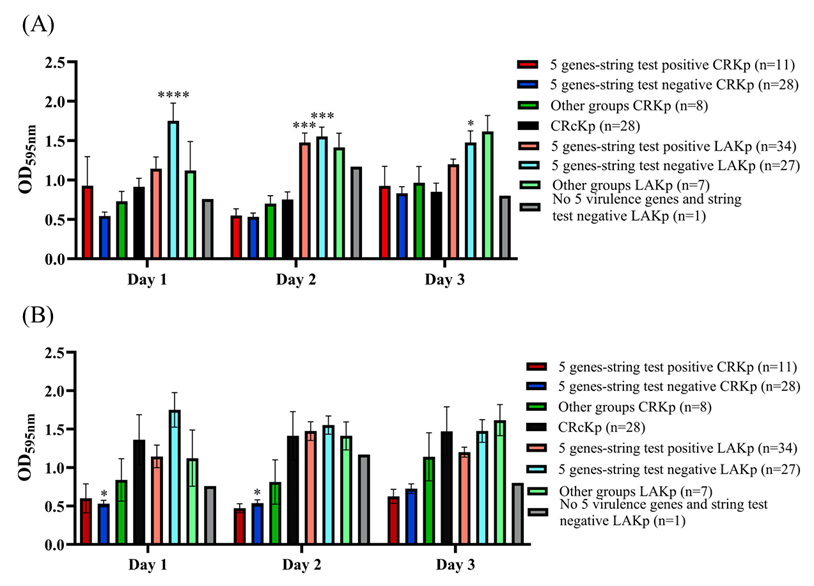


**Fig. S4**. **Biofilm formation of CRKp, CRcKp, and LAKp.** Biofilm formation of isolates in LB **(A)** and M9 **(B)** medium after one, two, and three days. The CRcKp group was used as the control group, and statistical analysis was conducted in comparison with this group. Differences among groups were analyzed using two-way ANOVA with Tukey’s multiple comparisons test. Statistical significance is indicated as follows: *, *p* < 0.05; ***, *p* < 0.001; ****, *p* < 0.0001.

**References**

1. Louden BC, Haarmann D, Lynne AM. 2011. Use of blue agar CAS assay for siderophore detection. Journal of microbiology & biology education 12:51-53.

2. Lee H, Baek JY, Kim SY, Jo H, Kang K, Ko J-H, Cho SY, Chung DR, Peck KR, Song J-H. 2018. Comparison of virulence between matt and mucoid colonies of Klebsiella pneumoniae coproducing NDM-1 and OXA-232 isolated from a single patient. Journal of Microbiology 56:665-672.
